# Three-Dimensional Human Cardiac Tissue Engineered by Centrifugation of Stacked Cell Sheets and Cross-Sectional Observation of Its Synchronous Beatings by Optical Coherence Tomography

**DOI:** 10.1155/2017/5341702

**Published:** 2017-02-22

**Authors:** Yuji Haraguchi, Akiyuki Hasegawa, Katsuhisa Matsuura, Mari Kobayashi, Shin-ichi Iwana, Yasuhiro Kabetani, Tatsuya Shimizu

**Affiliations:** ^1^Institute of Advanced Biomedical Engineering and Science, TWIns, Tokyo Women's Medical University, 8-1 Kawada-cho, Shinjuku-ku, Tokyo 162-8666, Japan; ^2^Panasonic Healthcare Co., Ltd., 2-38-5 Nishishinbashi, Minato-ku, Tokyo 105-8433, Japan; ^3^Panasonic Corporation, Osaka, Japan

## Abstract

Three-dimensional (3D) tissues are engineered by stacking cell sheets, and these tissues have been applied in clinical regenerative therapies. The optimal fabrication technique of 3D human tissues and the real-time observation system for these tissues are important in tissue engineering, regenerative medicine, cardiac physiology, and the safety testing of candidate chemicals. In this study, for aiming the clinical application, 3D human cardiac tissues were rapidly fabricated by human induced pluripotent stem (iPS) cell-derived cardiac cell sheets with centrifugation, and the structures and beatings in the cardiac tissues were observed cross-sectionally and noninvasively by two optical coherence tomography (OCT) systems. The fabrication time was reduced to approximately one-quarter by centrifugation. The cross-sectional observation showed that multilayered cardiac cell sheets adhered tightly just after centrifugation. Additionally, the cross-sectional transmissions of beatings within multilayered human cardiac tissues were clearly detected by OCT. The observation showed the synchronous beatings of the thicker 3D human cardiac tissues, which were fabricated rapidly by cell sheet technology and centrifugation. The rapid tissue-fabrication technique and OCT technology will show a powerful potential in cardiac tissue engineering, regenerative medicine, and drug discovery research.

## 1. Introduction

Recently, tissue engineering methodology and cell-based regenerative therapy have been progressing rapidly and are attracting attention worldwide [1–3]. Our laboratory has developed a scaffold-free tissue engineering methodology, cell sheet technology, with a temperature-responsive culture dish [4]. Confluently cultured cells are harvested from the dish as a continuous cell sheet by decreasing the culture temperature, which preserves the cell-cell junctions and extracellular matrix (ECM) [5]. A three-dimensional (3D) tissue can be easily engineered by stacking multiple cell sheets, and the resulting tissue with an intact ECM can be engrafted onto a target tissue efficiently without suturing [5–8]. Cell sheet transplantation improves the tissue functions in various animal models, and cell sheets have already been used clinically, and the feasibility of the therapy has been demonstrated [8]. Cell sheet stacking technology enables the transplantation of enormous numbers of cells in thicker 3D tissues and offers hope for more efficient therapies and a wider range of applications in regenerative medicine. Recently, we reported the rapid fabrication technique of a double-layered cell sheet-tissue by the combination of cell sheet stacking technology and centrifugation [9]. An optimal fabrication technique for thicker 3D tissue is important in tissue engineering and regenerative medicine to speed development of life-saving applications.

An efficient noninvasive cross-sectional observation system to study the structure and function of 3D tissues is also important in tissue engineering and regenerative medicine. Additionally, the observation system of an in vitro 3D human cardiac tissue model is important in the fields of cardiac physiology and the safety testing of candidate chemicals. However, the cross-sectional analysis of 3D tissue is difficult, while the analysis of two-dimensionally cultured cells is relatively easy. Optical coherence tomography (OCT) is a tomographic technology that uses innocuous near-infrared light, which is noncontact and noninvasive providing cross-sectional information of a living 3D tissue (to approximately 2 mm in depth; approximately 10 *μ*m in resolution) [10–12]. The safety and feasibility of OCT technology have been shown in various clinical applications. The resolution of OCT is significantly higher than other medical imaging technologies, such as intravascular ultrasound (IVUS): 100–200 *μ*m; standard computer tomography (CT) and magnetic resonance imaging (MRI): 500–1,000 *μ*m; and positron emission tomography (PET): 1,000–5,000 *μ*m [13]. Recently, we reported about an OCT system for observing cell sheet-tissues [14, 15].

In this report, a rapid fabrication technique of 3D tissue with a noninvasive observation technology was applied to the fabrication of a human cardiac tissue for aiming the clinical application. Specifically, cell-dense 3D human cardiac tissues were rapidly fabricated by stacking human induced pluripotent stem (iPS) cell-derived cardiac cell sheets with centrifugation; then the structure and function of the cell-layered cardiac tissues were observed by two OCT systems.

## 2. Materials and Methods

### 2.1. Human iPS Cell Culture, the Cardiac Differentiation, and Preparation of Cardiac Cell Sheet

Human iPS cells [201B7 stain (RBRC-HPS0001)] [16] were provided by the RIKEN BRC (Tsukuba, Japan) through the National Bio-Resources Project of the Ministry of Education, Culture, Sports, Science and Technology (MEXT), Japan, and cultured as shown in previous reports [17, 18]. In this study, human iPS cells were cultured and the cardiac differentiation was performed by a suspension culture with five factors [activin A, bone morphogenetic protein 4 (BMP-4), fibroblast growth factor-2 (FGF-2), vascular endothelial growth factor (VEGF), and Wnt-signaling antagonist, IWR-1] using a bioreactor system as shown in previous reports [17, 18]. Flow cytometry and immunocytochemistry showed that approximately 80% of the differentiated cells in the culture system were positive for cardiac related proteins (cardiac troponin T and sarcomeric *α*-actinin). The cardiac cells were seeded in 12-well UpCell® plates (CellSeed, Tokyo, Japan) (1.5 × 10^6^ cells/a well) and cultured at 37°C to harvest a cardiac cell sheet. After a 4-day cultivation, the culture dish with confluently cultured cardiac cells was transferred into a CO_2_ incubator set at 20°C for harvesting the cell sheet. After the transfer, the cardiac cells on the dish were spontaneously detached as a cardiac cell sheet within 60 min. Harvested cell sheets were layered on a 35 mm diameter polystyrene culture surface (Corning, NY, USA) [5, 6, 19], and layered cell sheets were centrifuged by a swing-type centrifuge (AllegraTM 21R Centrifuge) (Beckman Coulter, CA, USA) to accelerate their attachment as described in a previous report [9]. The movies of human iPS cell-derived cardiac cells were observed with a phase-contrast microscope, ET300 (Nikon, Tokyo, Japan), and the images were recorded with CCD camera equipment, acA1300-30uc (Basler, Ahrensburg, Germany) and the software StreamPix 6 (NorPix, Inc., Quebec, Canada).

### 2.2. Optical Coherent Tomography (OCT)

In this study two OCT systems [Proto 3(DT) (Panasonic Healthcare, Tokyo, Japan) [9, 14, 15, 20] and IVS-2000 (Santec, Aichi, Japan)] were used. Their specifications are summarized in Table 1. The observation of multilayered cell sheets was performed by the OCT system on a hot plate (37°C, ThermoPlus) (Tokai Hit, Shizuoka, Japan) at 0, 15, 30, 45, and 60 min with/without centrifugation. Those data were analyzed by the software ImageJ [21, 22]. The beatings of human iPS cell-derived cardiac cell sheets were recorded at 35 frames/second. Regions assessed as beatings within the cardiac cell sheets were the regions where the correlation of OCT signals at intervals of 0.09 s was lower than a predetermined level. The beating regions were labeled with green colors as shown in a previous report [14].

## 3. Results and Discussion

### 3.1. Observation of Single Human iPS Cell-Derived Cardiac Cell Sheet by OCT

**Figure 1:**
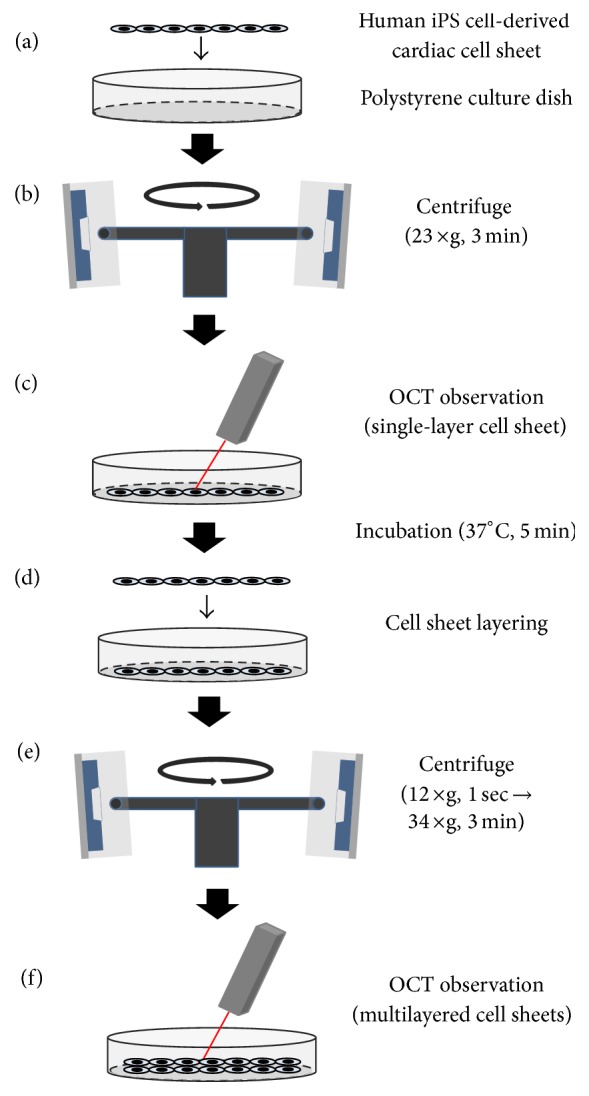
Fabrication and observation of layered human iPS cell-derived cardiac cell sheet-tissues using centrifugation. A detached human iPS cell-derived cardiac cell sheet was transferred onto a polystyrene culture dish (a). The culture dish with the cell sheet was centrifuged (23 ×g, 3 min), and the cross-sectional observation was performed by optical coherence tomography (OCT) (b and c). After incubation (37°C, 5 min), another cardiac cell sheet was transferred onto the first cardiac cell sheet (d). Multilayered cardiac cell sheets were centrifuged (12 ×g for 1 sec and again after removal of the extra medium 34 ×g for 3 min); then the cross-sectional observation of the cell sheets was performed (e and f).

**Figure 2:**
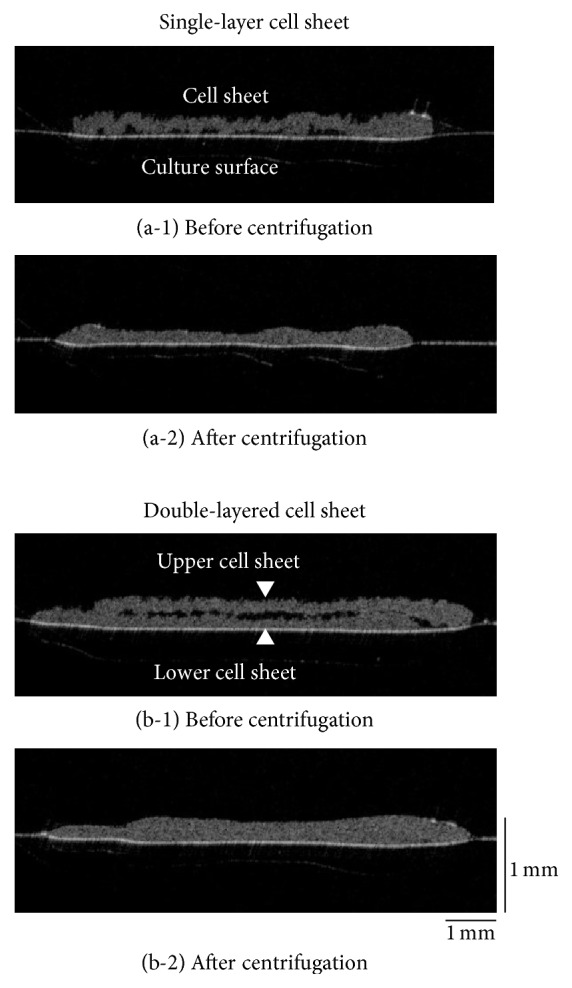
Observation of double-layered human iPS cell-derived cardiac cell sheets by optical coherence tomography (OCT). Just after the transfer of a human iPS cell-derived cardiac cell sheet onto a polystyrene culture dish, there were numerous spaces between the surfaces (a-1). After centrifugation, the spaces were almost entirely eliminated (a-2). Just after the transfer of a second cell sheet onto the first cell sheet, again there were numerous spaces between the surfaces (b-1). After centrifugation, the spaces were almost entirely eliminated (b-2). Three experiments were performed independently and all of them showed similar results. An OCT system [Proto 3(DT)] was used in this study.

Human iPS cell-derived cardiac cells on a temperature-responsive culture surface beat spontaneously and synchronously as shown in Supplementary Movie 1 in Supplementary Material available online at https://doi.org/10.1155/2017/5341702. After detachment, the cardiac cell sheet with medium was transferred onto a polystyrene culture dish and was spread out on the dish by three manipulations using a pipette: (i) rotation of the dish, (ii) slow aspiration, and/or (iii) gentle dropping of culture medium as shown in previous reports [5, 23], and thereafter, the medium was removed to promote attachment between the cell sheet and the culture surface (Figure 1(a)). Just after the transfer, many spaces were detected between them by OCT, even after the medium had been removed (Figure 2(a-1)). Then, the system clearly detected the beatings of the cardiac cell sheets and the transmission within the cell sheet cross-section (Supplementary Movie 2), suggesting that a detached cardiac cell sheet maintained the functional coupling. Next, the culture dish with the cell sheet was centrifuged (23 ×g for 3 min), and cross-sectional observation was performed by the OCT system (Figures 1(b) and 1(c)). Just after centrifugation for 3 min, the spaces between the culture dish and a cell sheet were mostly eliminated (Figure 2(a-2)). Importantly, the cell sheet beat spontaneously and robustly, and the transmission was detected as clearly before centrifugation as after centrifugation (Supplementary Movie 2). In a previous report, a C2C12 mouse myoblast sheet attached rapidly by the centrifugation method showed (i) active cell metabolism (glucose consumption and lactate production), which indicates the bioactivity of cells, (ii) the release of lactate dehydrogenase (LDH), which indicates cytotoxicity, and (iii) high production of VEGF, which is generally thought to be a major paracrine factor in the repair of the damaged heart tissue by myoblast sheet therapy [24], like those prepared by the conventional method [9]. Those results suggested cell sheets attached rapidly onto the dish by centrifugation without cell damage.

### 3.2. Observation of Multilayered Human iPS Cell-Derived Cardiac Cell Sheet by OCT

**Figure 3:**
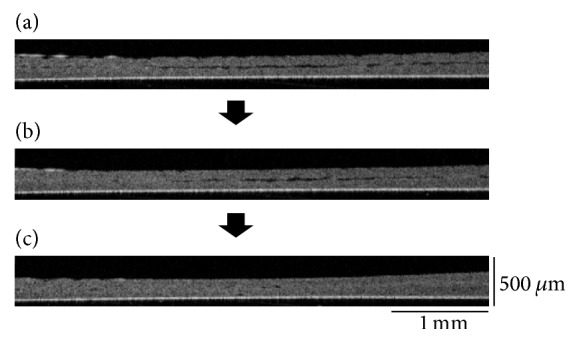
Optical coherence tomography (OCT) observation of double-layered human iPS cell-derived cardiac cell sheets fabricated by a conventional method. Just after the layering of a human iPS cell-derived cardiac cell sheet onto first cardiac cell sheet, there were significant spaces between the double-layered cell sheets (a). In the conventional method without centrifugation, some spaces between the double-layered cardiac cell sheets were detected even after the 15 min incubation (b), and spaces were hardly detected after the 30 min incubation (c). Three experiments were performed independently and all of them showed similar results. An OCT system (IVS-2000) was used in this study.

**Figure 4:**
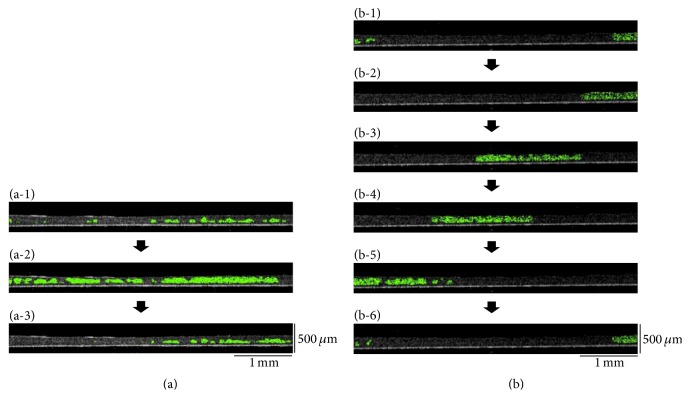
Observation of double-layered human iPS cell-derived cardiac cell sheets by optical coherence tomography (OCT). At 15 min cultivation after centrifugation, while the double-layered cardiac cell sheets did not continuously beat synchronously ((a-1) and (a-3)), the occasional synchronous beating was detected in the cell sheets (a-2). However, within 45 min cultivation, the cardiac cell sheets beat synchronously ((b-1)–(b-6)). Beating areas within the cell sheet are shown with green colors. The time-intervals between (a-1) and (a-3) or (b-1) and (b-6) were 4 s or 1 s, respectively. Three experiments were performed independently and all of them showed similar results. The other two double-layered cardiac cell sheets also synchronized within 45 min after centrifugation. An OCT system (IVS-2000) was used in this study.

Next, after incubation (37°C, 5 min), a second human iPS cell-derived cardiac cell sheet was layered onto the first cardiac cell sheet (Figure 1(d)). After centrifugation (12 ×g for 1 sec and after the remove of the extra medium 34 ×g for 3 min), the cross-sectional analysis of double-layered cardiac cell sheets was performed (Figures 1(e) and 1(f)). Just after layering, there were many spaces between the double-layered cell sheets even when the culture medium was removed (Figure 2(b-1)). After centrifugation for 3 min, most of the spaces were eliminated (Figure 2(b-2)). However, in a conventional method without centrifugation, some spaces between the double-layered cardiac cell sheets were detected even after the 15 min incubation, and the spaces were hardly detected after the 30 min incubation (Figure 3). After the transfer of a cardiac cell sheet onto a culture dish or the layering of two cardiac cell sheets and the removal of the culture medium, the culture dish with a cell sheet or layered cell sheets was incubated at 37°C for 30 min to wait for the strong attachment between the cell sheet and the culture dish or layered cell sheets and for avoiding the detachment by the manipulation of the spreading the cell sheet out and the cell sheet layering. Thus, double-layered cardiac cell sheets were fabricated in approximately 1 h by the conventional method for attachment between (i) the first cardiac cell sheet and the culture surface (30 min) and (ii) layered cardiac cell sheets (30 min). On the other hand, a double-layered cardiac cell sheet-tissue was fabricated in approximately 15 min by the centrifugation method, leading to significant time-saving (approximately 75%). Importantly, the cardiac cell-layered tissue beat even after centrifugation (Supplementary Movie 3), suggesting that the tissue was fabricated without cell damage. Additionally, the cross-sectional observation showed the synchronous beatings of the double-layered cardiac cell sheets within 45 min of cultivation after centrifugation (Figure 4). The electrical coupling of neighboring cardiomyocytes and the gap junction formation are established rapidly within 30 min after the cell-cell contact [25, 26]. Our previous reports also showed the rapid synchronization of layered cardiac cell sheets using neonatal rat cardiac cells [27, 28]. An electrophysiological analysis using a multiple-electrode extracellular action potential detection method showed the rapid electrical coupling of the layered cardiac cell sheets within one hour after layering. Additionally, connexin 43 was also rapidly detected between the layered cell sheets. Furthermore, a fluorescent dye transfer assay using calcein, which can pass through gap junctions [29], suggested rapid functional gap junction formation between the layered cell sheets. The rapid electrical coupling can be explained by the existence of connexin hemichannels, namely, gap junction precursors, on cell membranes [30–32]. It is thought that, after the cell-cell contact, rapid initial gap junction formation occurs via the assembly of the preexisting channel precursors without de novo cell biological reactions including RNA transcription, protein synthesis and maturation, and vesicular transport [30]. An immunohistological analysis showed that connexin 43 existed on the free cell membranes in a single cardiac cell sheet in addition to cell-to-cell interfaces [27]. Connexin 43 on the free cell membrane is thought to be gap junction precursors. The electrical coupling of layered cardiac cell sheets, which can be harvested without protease treatment, is thought to be established rapidly because the cell sheets have gap junction precursors already on the cell membranes and gap junctions at cell-to-cell interfaces. Deposited ECM on the cell sheet also may promote the initial adhesion between layered cardiac cell sheets and accelerate the assembly of the channel precursors. Connexin 43 was also detected at the edge of neighboring cardiomyocytes in a human iPS cell-derived cardiac cell sheet, and the electrical coupling of the cardiac cell sheet was shown by an electrophysiological analysis using the extracellular potential detection method [17, 33]. Additionally, the synchronous beatings and electrical coupling of layered cardiac cell sheets, which were fabricated by human iPS cell-derived cardiac cells or mouse embryonic stem (ES) cell-derived cardiac cells, were shown by the using of (i) phase-contrast microscopy, (ii) the electrophysiological analysis, and (iii) an intracellular calcium transient imaging method [17, 23, 34]. In this study, the rapid electrical coupling of layered human iPS cell-derived cardiac cell sheets, which were fabricated rapidly by centrifugation, was suggested by the cross-sectional observation of OCT.

**Figure 5:**
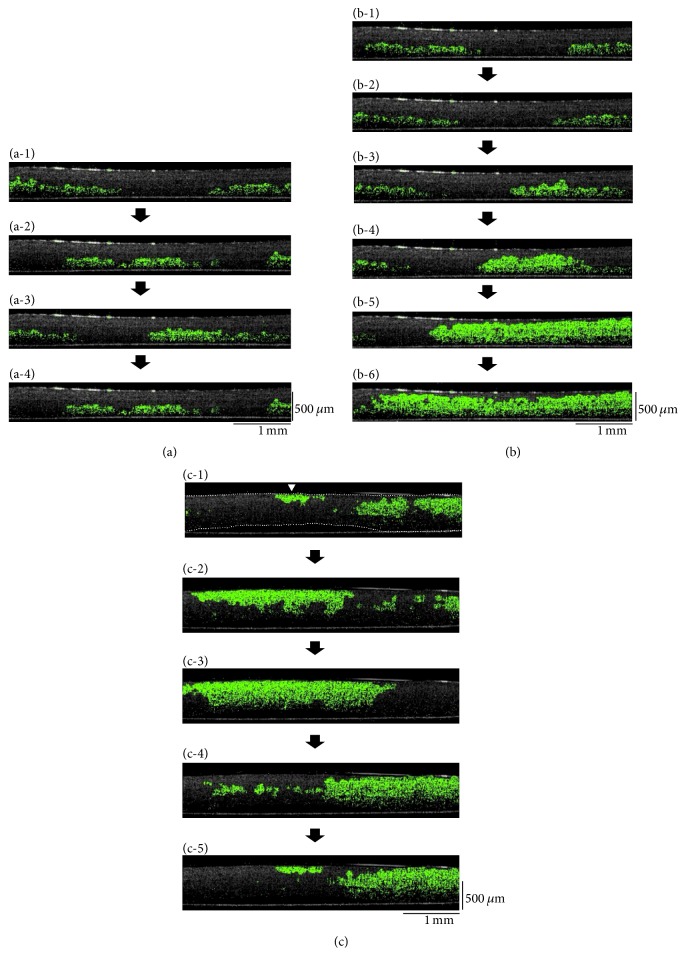
Observation of five-layered human iPS cell-derived cardiac cell sheets by optical coherence tomography (OCT). Just after layering of a fifth cardiac cell sheet onto the four-layered cardiac cell sheets and subsequent centrifugation, the lower cell sheets beat synchronously (a). The beating of the lower cardiac cell sheets was also occasionally transmitted to the upper cardiac cell sheets (b). Within 60 min after centrifugation, the five-layered cardiac cell sheets showed synchronous beatings (c). The time-intervals between (a-1) and (a-4), (b-1) and (b-6), or (c-1) and (c-5) were 1.6 s, 1 s, or 1 s, respectively. Beating areas within the cell sheet are shown with green colors. (c) The white arrowheads indicate the initiation sites of the beatings within each section of the tissue. In (c-1) the multilayered cardiac sheets are outlined with dashed lines. An OCT system (IVS-2000) was used in this study.

Next, five-layered human iPS cell-derived cardiac cell sheets were observed by OCT. Just after the layering of a fifth cardiac cell sheet onto a four-layered cardiac cell sheet and centrifugation, the lower cell sheets beat synchronously (Figure 5(a) and 5(b) and Supplementary Movie 4). The beating of the lower cardiac cell sheets also transmitted to the upper cardiac cell sheets occasionally, but not always. Within 60 min after the centrifugation, the five-layered cardiac cell sheets beat synchronously (Figure 5(c) and Supplementary Movie 4), suggesting complete functional coupling. Our previous report showed that in engineered tissues having more than four-layered cell sheets without vascular networks, they contained many damaged cells in damaged tissues caused by hypoxia/undernutrition, in the bottom areas directly in contact with the culture surface [35]. To prevent cell damage, multilayered cardiac cell sheets were detached from the dish and observed as shown in Figure 5(c), so that oxygen and nutrients could be supplied from both sides. These data showed that the cross-sectional transmission of the beatings within a multicell layered human cardiac tissue could be detected in real-time by OCT and suggested that the electrical coupling within the 3D cardiac cell sheet-tissue was intact.

Human ES cells [36] and human iPS cells [16, 37] are the focus of much new research in the fields of cell biology, tissue engineering, and regenerative medicine. Several clinical studies using stem cell-derived differentiated cells have already been started [38–40]. With the aim of clinical usage, human iPS cell-derived cardiac cell sheets were used in animal models, and these animal experiments demonstrated the safety and feasibility of the therapy [41, 42]. The transmission of rhythmical beatings of cardiac cells within a 3D cardiac tissue without arrhythmia is important for successful tissue fabrication. This study demonstrated a rapid fabrication technique for a 3D cardiac tissue using human iPS cell-derived cardiac cell sheets and centrifugation, and the synchronous beatings of multilayered cardiac cell sheet-tissue were detected noninvasively by an OCT system (Figures 4 and 5 and Supplementary Movies 3 and 4). In a previous report, cell sheets engrafted onto intensively beating porcine heart tissue were observed in real-time by OCT [43]. In the near future, human iPS cell-derived cardiac cell sheet-tissues will be used in clinical therapy for serious heart failure. In future clinical studies, the electrically and functionally coupled cardiac cell sheet-tissues fabricated by the rapid tissue-fabrication technique will be a useful tissue-engineered product for use in cardiac regenerative medicine. Additionally, the OCT system will contribute to the evaluation of (i) the structural and functional coupling of the engineered cardiac tissue and (ii) those of engrafted cardiac cell sheets on the target heart tissue.

Human cell tissues are highly desirable in the fields of cardiac physiology and safety testing for drug discovery research as well as regenerative medicine [44, 45]. It is generally thought that 3D cultured tissue much more closely resembles native in vivo situations compared to 2D cultured cells [46, 47]. A 3D human cardiac tissue is expected to be used as an in vitro model for detecting proarrhythmia drugs. Presently, although the cost of drug discovery research has been increasing, the number of new drugs approved and launched into the market is decreasing steadily. For example, in 2001, while 30% of clinically tested drugs were abandoned because of a lack of efficacy, another 30% were also abandoned because of safety concerns such as cardiotoxicities [48, 49]. In this study, the cross-sectional transmission of cardiac cell-beatings within a human cardiac tissue was clearly detected by OCT (Figures 4 and 5 and Supplementary Movies 3 and 4). Additionally, the quantitative analysis of the conduction velocity is also important and interesting in the fields of cardiac physiology and cardiotoxicity testing as well as cardiac tissue engineering and regenerative medicine. At present, we are preparing electrophysiological analyses as well as OCT observation using multilayered human iPS cell-derived cardiac cell sheets and several drugs including ion channel inhibitors. Additionally, we are also preparing to establish the quantitative assessment system of the conduction velocity in layered human iPS cell-derived cardiac cell sheet-tissues. Those systems will contribute to a better understanding of cardiac physiology and be useful in the detection of cardiotoxic drugs including arrhythmogenicity.

## 4. Conclusion

A 3D human cardiac tissue was fabricated rapidly by a combination of cell sheet stacking technique and centrifugation for aiming the clinical usage. The structure and beating of 3D human cardiac tissues were observed cross-sectionally in real-time by OCT. The observation showed the synchronous beatings of the multilayered human cardiac cell tissues. The rapid tissue-fabrication technique and OCT technology will contribute to a better understanding of cardiac physiology and lead to future advances in cardiac tissue engineering, regenerative medicine, and safety testing for drug discovery research.

## Supplementary Material

Supplementary Movie 1: Observation of human iPS cell-derived cardiac cells by microscopy. The movie shows human iPS cell-derived cardiac cells on a temperature-responsive culture surface. The magnification: x100.
Supplementary Movie 2: Observation of a single-layer human iPS cell-derived cardiac cell sheet by optical coherence tomography (OCT). The movie shows the cross-section of a beating human iPS cell-derived cardiac cell sheet on a polystyrene culture surface just after transfer and centrifugation. Beating areas within the cell sheet are shown with green colors. Although just after transfer (Just after transfer) there were many spaces between the cell sheet and the culture surface, after centrifugation (Just after centrifugation) the spaces were hardly observed by OCT. The movie shows the beating phenomenon at real-time speed. An OCT system (IVS-2000) was used in this study.
Supplementary Movie 3: Observation of double-layered human iPS cell-derived cardiac cell sheets by optical coherence tomography (OCT) at 15 min (Before synchronization) and 45 min (After synchronization) after centrifugation. The movie shows the cross-sectional observation of double-layered human iPS cell-derived cardiac cell sheets. Beating areas within the cell sheet are shown with green colors. The movie shows the phenomenon at real-time speed. An OCT system (IVS-2000) was used in this study.
Supplementary Movie 4: Observation of five-layered human iPS cell-derived cardiac cell sheets by optical coherence tomography (OCT) just after the last centrifugation (Before synchronization) and at 60 min (After synchronization). The movie shows the cross-sectional observation of five-layered human iPS cell-derived cardiac cell sheets. Beating areas within the cell sheet are shown with green colors. The movie shows the phenomenon at real-time speed. An OCT system (IVS-2000) was used in this study.

## Figures and Tables

**Table 1:** Specifications of two OCT systems.

OCT system	Cutting (slice) direction	Area	Resolution	Observation
Proto 3(DT)	Vertical	Horizontal: 14 × 14 mm Vertical: 2.3 mm	Horizontal: 20 *μ*m Vertical: 9 *μ*m	Attachment between human cardiac cell sheets (Figure 2)
IVS-2000		Horizontal: 6.5 × 6.5 mm Vertical: 3.9 mm	Horizontal: 8.7 *μ*m Vertical: 8.7 *μ*m	Beatings of human cardiac cell sheets (Figures 3–5 and Supplementary Movies 2–4)
